# Monocyte–Lymphocyte Ratio and Dysglycemia: A Retrospective, Cross-Sectional Study of the Saudi Population

**DOI:** 10.3390/healthcare10112289

**Published:** 2022-11-15

**Authors:** Mohammad A. Alfhili, Jawaher Alsughayyir, Ahmed M. Basudan, Roua Alsubki, Saleh Alqahtani, Zuhier A. Awan, Mohammed R. Algethami, Yazeed A. Al-Sheikh

**Affiliations:** 1 Department of Clinical Laboratory Sciences, College of Applied Medical Sciences, King Saud University, Riyadh 12372, Saudi Arabia; 2 King Faisal Medical City for Southern Regions, Abha 62527, Saudi Arabia; 3 Department of Clinical Biochemistry, Faculty of Medicine, King Abdulaziz University, Jeddah 21589, Saudi Arabia; 4 Department of Clinical Pathology, Al-Borg Medical Laboratories, Jeddah 23437, Saudi Arabia; 5 Preventive Medicine and Public Health, Ministry of Health, Jeddah 23325, Saudi Arabia

**Keywords:** diabetes, monocyte–lymphocyte ratio, biomarker, Saudi Arabia

## Abstract

Background: Abnormalities in fasting blood glucose (FBG) resulting in hypoglycemia (OG), impaired fasting glycemia (IFG), or hyperglycemia (HG) arise from disordered metabolic regulation caused in part by inflammation. To date, there is a dearth of evidence regarding the clinical utility of the monocyte–lymphocyte ratio (MLR), an emerging inflammatory index, in the management of dysglycemia. Methods: This retrospective, cross-sectional study explored MLR fluctuations as a function of glycemic control in 14,173 Saudi subjects. Data collected from 11 August 2014 to 18 July 2020 were retrieved from Al-Borg Medical Laboratories. Medians were compared by Mann–Whitney U or Kruskal–Wallis tests and the prevalence, relative risk (RR), and odds ratio (OR) were calculated. Results: MLR was significantly elevated in IFG (*p* < 0.0001) and HG (*p* < 0.05) groups compared to the normoglycemia (NG) group, and individuals with elevated MLR (>0.191) had significantly increased FBG (*p* < 0.001). The risk of IFG (RR = 1.12, 95% CI: 1.06–1.19, *p* < 0.0002) and HG (RR = 1.10, 95% CI: 1.01–1.20, *p* < 0.0216) was significantly increased if MLR was elevated, and individuals with elevated MLR were 1.17 times more likely to have IFG (OR = 1.17, 95% CI: 1.08–1.26, *p* < 0.0002) and 1.13 times more likely to have HG (OR = 1.13, 95% CI: 1.02–1.24, *p* < 0.0216). Conclusion: Elevated MLR is correlated with and carries a greater risk for IFG and HG. However, large prospective cohort studies are needed to establish the temporal relationship between MLR and FBG and to examine the prognostic value of this novel marker.

## 1. Introduction

Maintenance of plasma glucose levels requires complex metabolic regulation involving insulin, glucose uptake, glycogenesis, gluconeogenesis, glucagon, and glycogenolysis. Hypoglycemia (OG) is most often iatrogenic in nature, with type I diabetes mellitus (T1DM) patients being at a higher risk than those with T2DM. Other causes, however, include alcohol consumption, critical illness, and non-islet cell tumors [1]. On the other hand, disturbances in glucose homeostasis resulting in impaired fasting glycemia (IFG) or hyperglycemia (HG) often arise secondary to obesity, aging, fat- and carbohydrate-rich diets, sedentary lifestyle, or genetic predisposition. In these cases, increased fasting blood glucose (FBG) is associated with an inflammatory state characterized by insulin resistance, cytokine release, fatty acid exhaustion, mitochondrial dysfunction, and endoplasmic reticulum stress [2].

It has been demonstrated that macrophages and lymphocytes accumulate in the milieu of adipocytes [3], highlighting a central role of immune cells in obesity-induced and adipose tissue inflammation. In particular, mounting evidence suggests that under HG monocytes upregulate the expression of toll-like receptors (TLR) 2 and TLR4 and the release of IL-1β, IL-6, IL-18, and TNF-α, albeit through ambiguous mechanisms [4]. This further precipitates inflammatory and oxidative damage and predisposes to vascular and neurological complications typical of DM. This is underscored by studies reporting that monocytes are highly abundant in atherosclerotic lesions of DM [5], display a persistent M1 inflammatory phenotype [6], and that the Ly6C^hi^ phenotype is required for dissolution of lesion [7]. Furthermore, lymphocyte counts have been demonstrated to be lower with altered metabolism and impaired response in diabetics compared to non-diabetics [8,9,10]. Similarly, the distribution of naïve CD4^+^, Th1, Th17, and Treg cells in T2DM shifts toward promoting a hyperactive immune response with concurrent inflammation [11]. 

The monocyte–lymphocyte ratio (MLR) is a simple, inexpensive, and reproducible index of monocyte and lymphocyte counts in peripheral blood. Monocytosis, or elevated monocyte count, is clinically significant in a wide range of conditions including acute and chronic infections, tumors, auto-inflammatory disorders, iatrogenesis, and non-specific stress [12]. Lymphopenia, or reduced lymphocyte count, is seen in severe combined immunodeficiency [13], autoimmune disorders such as systemic lupus erythematosus [14], T2DM [15], end-stage renal disease [16], tumors [17], iatrogenesis, and viral and bacterial infections [18,19]. Since inflammation is a common denominator in the constellation of conditions affecting peripheral monocytes and lymphocytes, elevated MLR caused by increased monocyte and/or reduced lymphocyte counts has frequently been used as an inflammatory marker in varying clinical contexts [20]. For instance, increased MLR was observed in non-affective psychosis [21], coronary artery disease (CAD) [22], preeclampsia [23], and stroke-associated pneumonia [24], among others. Of note, MLR has also been found to be a prognostic marker in tuberculosis [25] and cancer [26]. To date, reports on the relationship between MLR and glycemic status remain severely lacking, and this work, thus, aims to examine the association between MLR and FBG levels using a large, population-based approach.

## 2. Materials and Methods

### 2.1. Data Collection and Study Design

**Figure 1:**
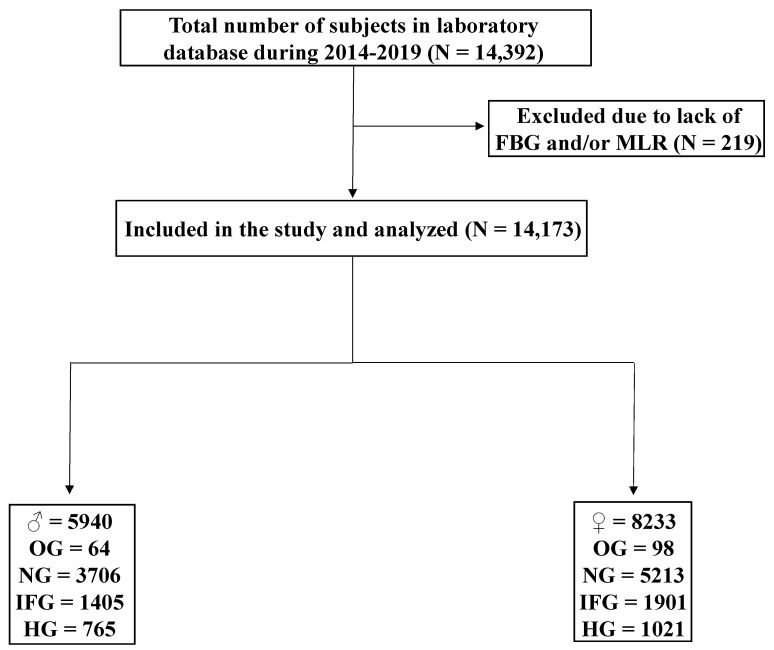
A flow chart of study design.

This is a retrospective, cross-sectional study of MLR fluctuations as a function of FBG and HbA1c in a total of 14,173 subjects (Figure 1). The study protocol was approved by the Biomedical Ethics Unit of Al-Borg Medical Laboratories; the legal owner of the data, under approval #07/21. Age, sex, and laboratory results were collected from 11 August 2014 to 18 July 2020, and no informed consent was required given the retrospective nature of the study and the lack of access to personal identifiable information. Subjects were stratified based on the ADA guidelines [27,28] for FBG into OG (FBG < 70 mg/dL), normoglycemia (NG; 70–99 mg/dL), IFG (100–125 mg/dL), and HG (≥126 mg/dL) groups. Subjects were considered normal if HbA1c was <5.7%, pre-diabetic if 5.7–6.4%, and diabetic if ≥6.5% [28]. To validate HbA1c results, anemia was excluded as defined by a hemoglobin level of <12 g/dL [29]. MLR of >0.191 was considered high based on the best cutoff (i.e., highest sensitivity and specificity) as revealed by receiver operating characteristics (ROC) curve analysis.

### 2.2. Statistical Analysis

The data were not normally distributed as revealed by D’Agostino and Pearson test and Kolmogorov–Smirnov test (*p* < 0.0001) and hence nonparametric tests were used for statistical analysis. Two groups were analyzed by Mann–Whitney U test while three or more were analyzed by Kruskal–Wallis test followed by Dunn’s multiple comparisons test. Figures show medians ± interquartile range (IQR). Association between two variables was determined by Spearman’s correlation and by calculations of the relative risk (RR) and odds ratio (OR). Sensitivity and specificity were examined by ROC curve analysis and area under the curve (AUC) determination. GraphPad Prism v9.2.0 (GraphPad Software, Inc., San Diego, CA, USA) was used for statistical analysis, and the cutoff for significance was set at a *p* value of <0.05.

## 3. Results

### 3.1. MLR Is Significantly Elevated in IFG and HG

**Figure 2:**
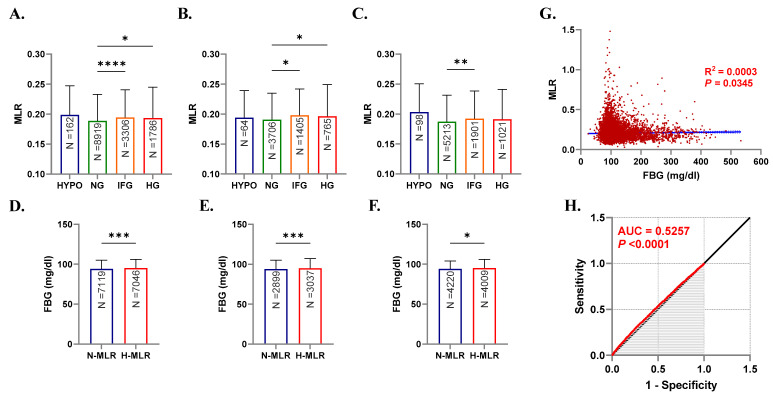
Patterns of MLR in light of FBG levels. Medians + IQR of MLR in both genders (**A**), males (**B**), and females (**C**), and of FBG in both genders (**D**), males (**E**), and females (**F**). Spearman’s correlation (**G**) and ROC curve (**H**) of MLR and FBG. ns indicates not significant, while * *p* < 0.05, ** *p* < 0.01, *** *p* < 0.001, and **** *p* < 0.0001.

As shown in Figure 2A, MLR was significantly increased in IFG (0.194, 0.158–0.241, *p* < 0.0001) and HG (0.193, 0.155–0.244, *p* < 0.05) groups in comparison to the NG group (0.189, 0.154–0.233). This was also true when males were considered alone with a significant increase in MLR (*p* < 0.05) from the NG group median of 0.191 (0.155–0.235) to 0.198 (0.160–0.242) and 0.196 (0.159–0.249) in IFG and HG groups, respectively (Figure 2B). In females, only the increase in MLR of the IFG group attained statistical significance compared to the control NG group (0.192, 0.158–0.239 vs. 0.187, 0.152–0.232, *p* < 0.01) as revealed in Figure 2C. 

These findings highlight the gender disparity observed in DM which could be, at least in part, explained by sex-specific monocyte physiology and differential sex steroid availability and its influence on insulin sensitivity and glucose homeostasis [12,30].

### 3.2. FBG Is Significantly Increased in Individuals with Elevated MLR

Figure 2D shows that the median FBG in subjects with normal MLR (N-MLR) significantly increased (*p* < 0.001) from 94.0 (87.0–105) to 95.0 (87.0–106) in the high MLR (H-MLR) group. Both male and female subjects exhibited the same pattern of increase (94.0, 87.0–105 vs. 95.0, 88.0–107, *p* < 0.001) and (94.0, 87.0–104 vs. 95.0, 87.0–106, *p* < 0.05) as seen in Figure 2E,F, respectively. 

Stratified by MLR, FBG is consistently elevated in H-MLR irrespective of gender which propounds a state of systemic inflammation synchronous with glucose build up.

### 3.3. Correlation between MLR and FBG

Although our simple linear regression model shown in Figure 2G found no relationship between MLR and FBG, the two variables seem to be related but fluctuations in FBG cannot be exclusively explained by those in MLR (R^2^ = 0.0003, *p* < 0.0345). Analysis of the ROC curve indices (Figure 2H) revealed an AUC of 0.5257 (*p* < 0.0001) reflecting poor diagnostic accuracy of MLR for HG. 

Several factors may have been involved in the significant, but indirect association between MLR and FBG, most notably other inflammatory mediators, such as cytokines, fetuin-A, chemerin, and vaspin which lead to β-cell failure and insulin resistance [31]. The requirement of these factors for the observed association between MLR and FBG must be established in future studies. 

### 3.4. Differential Influence of Age and Gender on MLR 

**Figure 3:**
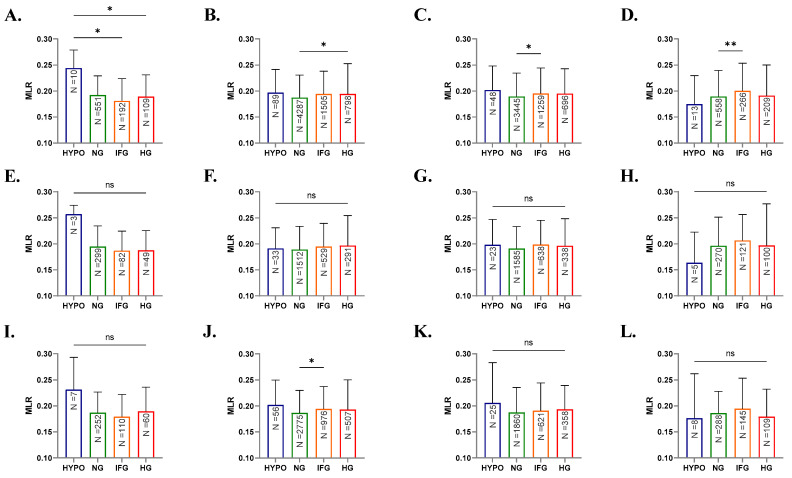
Effect of age, sex, and FBG on MLR. Medians + IQR of MLR in young, young adults, adults, and elderlies of both genders (**A**–**D**), of males (**E**–**H**), and of females (**I**–**L**). ns indicates not significant, while * *p* < 0.05 and ** *p* < 0.01.

Further stratification of subjects by age revealed distinct and shared patterns in MLR fluctuations. In Figure 3A, young subjects of both genders showed significantly reduced MLR in IFG (0.181, 0.147–0.224, *p* < 0.05) and HG (0.189, 0.149–0.231, *p* < 0.05) groups compared to the OG group (0.244, 0.210–0.230). Compared to the NG group, significant increases in MLR were seen in young adults of the HG group (0.188, 0.153–0.231 vs. 0.195, 0.155–0.253, *p* < 0.05), and adults (0.189, 0.155–0.235 vs. 0.195, 0.159–0.244, *p* < 0.05) and elderlies of the IFG group (0.189, 0.155–0.239 vs. 0.20, 0.168–0.253, *p* < 0.05) as depicted in Figure 3B–D. When either males or females were analyzed alone, no significant differences in MLR were found among the different glycemic states except in female young adults (Figure 3J) who exhibited significant elevation in MLR from 0.186 (0.151–0.230) in the NG group to 0.194 (0.157–0.237) in the IFG group (*p* < 0.05).

Altogether, these results support the influence of age on glycemic control with older groups of both genders exhibiting significant MLR elevations in IFG and HG. Further, MLR is a more sensitive marker in adults and elderlies as it was significantly elevated in IFG in these groups. Age-related differences in immune regulation resulting in an aggravated inflammatory response and energy depletion by lymphocytes [32,33] may account for this observation.

### 3.5. Elevated MLR Is Associated with Increased Risk of IFG and HG

Analysis of the overall and within-group prevalence of H-MLR in the study population showed that it was less prevalent in the NG group and more prevalent in the OG, IFG, and HG groups with increases of 28.0%, 9.39%, and 5.40%, respectively (Table 1). Moreover, as shown in Table 2, elevated MLR was associated with increased risk of IFG (RR = 1.12, 95% CI: 1.06–1.19, *p* < 0.0002) and HG (RR = 1.10, 95% CI: 1.01–1.20, *p* < 0.0216). Additionally, elevated MLR was 1.17 times more likely to fall into the IFG group (OR = 1.17, 95% CI: 1.08–1.26, *p* < 0.0002) and 1.13 times more likely to suffer from HG (OR = 1.13, 95% CI: 1.02–1.24, *p* < 0.0216).

Collectively, these findings indicate that increased MLR predisposes to IFG and HG, possibly due to the inflammatory potential of monocytes.

### 3.6. MLR Is Not Influenced by HbA1c Levels

**Figure 4:**
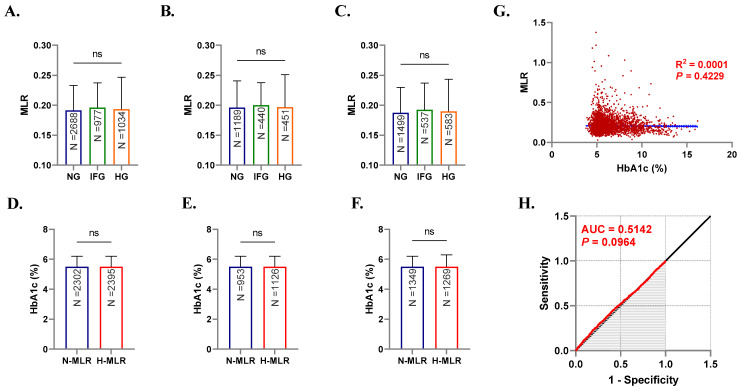
Patterns of MLR in light of HbA1c levels. Medians + IQR of MLR in both genders (**A**), males (**B**), and females (**C**), and of HbA1c in both genders (**D**), males (**E**), and females (**F**). Spearman’s correlation (**G**) and ROC curve (**H**) of MLR and HbA1c. ns indicates not significant.

Unlike FBG, examination of MLR in light of HbA1c levels found no variance or association between the two parameters (Figure 4A–H). While HbA1c reflects long-term (2–3 months) glycemic control, interindividual variation in this indicator may have accounted for its lack of association with MLR. Variables other than FBG, such as gender, red cell turnover, blood acidity, and race, have been described as contributing factors [34] highlighting the discord between HbA1c and FBG.

## 4. Discussion

Inexpensive, quick, and reproducible indices are highly desired in clinical practice. In this work, we show, for the first time, that increased MLR is associated with IFG and HG in the Saudi population. We have recently reported that the neutrophil–lymphocyte ratio (NLR) is similarly elevated and more prevalent in hyperglycemic Saudis [27] which suggests concurrent systemic inflammation. MLR, on the other hand, is also significantly raised in IFG (Figure 2A–C) making it a more sensitive marker of glucose status compared to NLR. Likewise, C-reactive protein (CRP) is superior to NLR in discriminating prediabetics [35,36], which points to a possible advantage in assessing both MLR and CRP in DM management.

The Saudi Abnormal Glucose Metabolism and Diabetes Impact Study (SAUDI-DM) revealed that IFG and DM affect 22.6% and 11.9% of the Saudi population, respectively [37]. Stratified by MLR, our study found that subjects demonstrated significantly elevated FBG with increased MLR compared to those with normal MLR (Figure 2D–F). In contrast, we found no relation between MLR and HbA1c (Figure 4). The differential impact of NLR on HbA1c was also investigated in Brazilian and Turkish diabetics and no relation was established [38,39]. Since HbA1c reflects long-term glycemic control, it seems plausible to assume that it is less susceptible to transient or acute inflammatory stimuli which may explain the lack of association with NLR and MLR in this and in other various reports.

The value of MLR in the management of DM extends to common complications despite its poor diagnostic performance (Figure 2G,H). In diabetic kidney injury, high MLR was correlated with microalbuminuria and was thus a predictive marker for diabetic nephropathy [40,41]. MLR was similarly elevated in proliferative diabetic retinopathy and may thus have a prognostic value [42] in combination with fibrinogen levels [43]. In contrast, Ilhan et al. demonstrated that NLR but not MLR was significantly elevated in diabetic macular edema [44]. Accordingly, identifying the clinical contexts in which MLR offers a prognostic value for DM complications must be the focus of future studies.

Interestingly, when stratified by both age and gender, males exhibited no significant changes in FBG levels relative to MLR (Figure 3E–H). In DM, there exists a gender disparity in the predisposition, prevalence, and disease progression among patients. For instance, men demonstrate an earlier peak in DM prevalence than women [45], and in the Middle East, DM is more common in females than in males [46]; an observation in opposition to the vast majority of the world. Since estrogens are essential for carbohydrate and lipid utilization, sex steroids may play a protective role against DM development as highlighted by the diminished incidence of DM in menopausal women on estrogen therapy [47]. Nevertheless, large, longitudinal studies are needed to characterize sex-specific determinants of DM susceptibility and development.

Evidence from a meta-analysis points to an inflammatory basis for non-affective psychoses manifested as increased MLR in bipolar disorder, major depressive disorder, and schizophrenia [21]. A variety of cells in the brain are differentiated from monocytes including macrophages in the meninges and choroid plexus and perivascular cells. These cells act in tandem with microglia in neuropathological conditions directed by inflammatory cytokines which also promote infiltration of the brain tissue with circulating monocytes [48,49].

Regulated fluctuations in white blood cell counts are essential to produce a modest inflammatory state that maintains successful pregnancy [50]. In preeclampsia, an exaggerated immune activation mediated through cytokines released from placental tissue precipitates macrophage deposition in the placenta leading to TNF-α-induced apoptosis of trophoblasts [23]. Moreover, it has been demonstrated that lymphocyte proliferation is inhibited in preeclampsia [51], which may explain the increased MLR in these patients. Recently, elevated MLR was found to predict unfavorable outcomes in pregnant women with HG [52].

MLR has also been investigated in the context of cardiovascular disease by several groups. In CAD, it has been shown that MLR could be useful as an independent risk factor and as a predictor of CAD severity with superior performance than NLR [22]. This was also confirmed in another study which found that MLR was an independent predictor of long-term major adverse cardiac events in myocardial infarction patients [53]. A significant correlation between MLR and the length of hospital stay was also reported in myocarditis patients [54]. Cheng et al. have recently shown that elevated MLR was associated with the development of stroke-associated pneumonia and therefore may be exploited for earlier intervention [24]. Moreover, MLR and NLR were independent predictors of saphenous vein graft disease in patients undergoing coronary artery bypass grafting [55].

In arthritis, MLR was significantly elevated during gout attacks and may thus serve as a predictive marker for the attack [56]. However, although increased MLR was associated with severe tuberculosis, it failed to predict response to treatment or mortality [57]. Congruently, MLR was not significantly altered in COVID-19 patients [58]. In dialysis patients with increased MLR, the risk of cardiovascular events and length of infectious disease hospitalization was significantly increased compared to those with normal MLR [59]. Notably, in a retrospective study of a very large cohort of critically ill patients, increased MLR was associated with an increased risk of mortality, the need for continuous renal replacement therapy, mechanical ventilation, and hospitalization [60]. 

MLR has also gained interest as a clinical aid in oncology as it was found to be significantly elevated in lymphoma [61] and ovarian cancer [62]. In the latter, calculating MLR prior to surgical intervention could be of clinical value in predicting stage, grade, and metastasis. Additionally, MLR was associated with serum CA-125 levels in a retrospective, cross-sectional study of Nigerian ovarian cancer patients [63]. Furthermore, MLR performed better than NLR in predicting the progression and overall survival of bladder cancer patients [64,65,66,67], and was invaluable for the prognosis and survival of cervical cancer and non-small-cell lung cancer patients [68,69]. Likewise, MLR was better than NLR in diagnosing colorectal cancer [70] and complemented prostate-specific antigen in predicting prostate cancer and in reducing false positive results [71].

The current report also demonstrates that IFG and HG are more prevalent in H-MLR compared to N-MLR subjects (Table 1) and that elevated MLR may be a risk factor for IFG and HG (Table 2), highlighting the potential role of MLR to predict and monitor the glycemic status in Saudis. In congruence, MLR was associated with the prevalence of proliferative diabetic retinopathy in an American population [42], and reduced monocyte counts were associated with retinopathy in diabetic Chinese subjects [72]. Indeed, causation remains to be interrogated in longitudinal studies.

Our study has numerous advantages, most notably the very large sample size and the streamlined generation, acquisition, and recordkeeping of laboratory data. Limitations, however, include the inability to determine the causality and temporal relation between FBG and MLR given the cross-sectional design of the study, and the unavailability of sufficient potential confounding variables including lifestyle habits, comorbidities, and medication intake.

## 5. Conclusions

In conclusion, our study identifies MLR as a novel, inexpensive, and reproducible biomarker in the pre-diabetic and diabetic Saudi population. Prospective studies must examine why FBG, but not HbA1c, is influenced by MLR fluctuations in addition to investigating other glycemic and inflammatory markers, such as insulin, glycated albumin, fructosamine, and gasdermins. Longitudinal studies are also needed to assess the potential impact of anthropometric variables on MLR and glycemic control.

## Figures and Tables

**Table 1:** Prevalence of glycemic disturbances relative to MLR.

	OG	NG	IFG	HG
**Overall**	1.14	62.92	22.98	12.94
**N-MLR**	0.50	32.47	10.97	6.30
**H-MLR**	0.64	30.45	12.00	6.64
**Within-group**				
**N-MLR**	43.82	51.60	47.77	48.66
**H-MLR**	56.17	48.39	52.22	51.33

OG, hypoglycemia; NG, normoglycemia; IFG, impaired fasting glycemia; HG, hyperglycemia; N-MLR, normal MLR; H-MLR, high MLR.

**Table 2:** Risk assessment of elevated MLR.

	Score	95% CI	*z* Statistic	*P*
**RR**				
**OG**	1.36	0.99 to 1.85	1.96	0.0505
**IFG**	1.12	1.06 to 1.19	3.74	**0.0002**
**HG**	1.10	1.01 to 1.20	2.30	**0.0216**
**OR**				
**OG**	1.37	0.99 to 1.87	1.96	0.0504
**IFG**	1.17	1.08 to 1.26	3.74	**0.0002**
**HG**	1.13	1.02 to 1.24	2.30	**0.0216**

OG, hypoglycemia; IFG, impaired fasting glycemia; HG, hyperglycemia.

## Data Availability

Data is available from the corresponding author upon reasonable request, and with permission of Al-Borg Medical Laboratories.

## References

[1] Koch C.A., Petersenn S. (2018). Black swans—Neuroendocrine tumors of rare locations. Rev. Endocr. Metab. Disord..

[2] Bano G. (2013). Glucose homeostasis, obesity and diabetes. Best Pract. Res. Clin. Obstet. Gynaecol..

[3] Pond C.M. (2005). Adipose tissue and the immune system. Prostaglandins Leukot. Essent. Fat. Acids.

[4] Kanter J.E., Hsu C.C., Bornfeldt K.E. (2020). Monocytes and Macrophages as Protagonists in Vascular Complications of Diabetes. Front. Cardiovasc. Med..

[5] Burke A.P., Kolodgie F.D., Zieske A., Fowler D.R., Weber D.K., Varghese P.J., Farb A., Virmani R. (2004). Morphologic findings of coronary atherosclerotic plaques in diabetics: A postmortem study. Arterioscler. Thromb. Vasc. Biol..

[6] Yan J., Tie G., Wang S., Tutto A., DeMarco N., Khair L., Fazzio T.G., Messina L.M. (2018). Diabetes impairs wound healing by Dnmt1-dependent dysregulation of hematopoietic stem cells differentiation towards macrophages. Nat. Commun..

[7] Rahman K., Vengrenyuk Y., Ramsey S.A., Vila N.R., Girgis N.M., Liu J., Gusarova V., Gromada J., Weinstock A., Moore K.J. (2017). Inflammatory Ly6Chi monocytes and their conversion to M2 macrophages drive atherosclerosis regression. J. Clin. Investig..

[8] Bailin S.S., McGinnis K.A., McDonnell W.J., So-Armah K., Wellons M., Tracy R.P., Doyle M.F., Mallal S., Justice A.C., Freiberg M.S. (2020). T Lymphocyte Subsets Associated With Prevalent Diabetes in Veterans With and Without Human Immunodeficiency Virus. J. Infect. Dis..

[9] Otton R., Mendonca J.R., Curi R. (2002). Diabetes causes marked changes in lymphocyte metabolism. J. Endocrinol..

[10] Muniyappa R., Gubbi S. (2020). COVID-19 pandemic, coronaviruses, and diabetes mellitus. Am. J. Physiol. Endocrinol. Metab..

[11] Xia C., Rao X., Zhong J. (2017). Role of T Lymphocytes in Type 2 Diabetes and Diabetes-Associated Inflammation. J. Diabetes Res..

[12] Mangaonkar A.A., Tande A.J., Bekele D.I. (2021). Differential Diagnosis and Workup of Monocytosis: A Systematic Approach to a Common Hematologic Finding. Curr. Hematol. Malig. Rep..

[13] Aluri J., Gupta M.R., Dalvi A., Mhatre S., Kulkarni M., Desai M., Shah N.K., Madkaikar M.R. (2019). Lymphopenia and Severe Combined Immunodeficiency (SCID)—Think Before You Ink. Indian J. Pediatr..

[14] Martin M., Guffroy A., Argemi X., Martin T. (2017). Systemic lupus erythematosus and lymphopenia: Clinical and pathophysiological features. Rev. Med. Interne.

[15] Cheng Y., Yue L., Wang Z., Zhang J., Xiang G. (2021). Hyperglycemia associated with lymphopenia and disease severity of COVID-19 in type 2 diabetes mellitus. J. Diabetes Complicat..

[16] Lepe-Zuniga J.L., Morales-Molina P., Garcia-Nandayapa G.A. (2016). End stage renal disease lymphopenia; characterization and clinical correlation. Rev. Médica Del Inst. Mex. Del Seguro Soc..

[17] Tang C., Liao Z., Gomez D., Levy L., Zhuang Y., Gebremichael R.A., Hong D.S., Komaki R., Welsh J.W. (2014). Lymphopenia association with gross tumor volume and lung V5 and its effects on non-small cell lung cancer patient outcomes. Int. J. Radiat. Oncol. Biol. Phys..

[18] Guo Z., Zhang Z., Prajapati M., Li Y. (2021). Lymphopenia Caused by Virus Infections and the Mechanisms Beyond. Viruses.

[19] Turett G.S., Telzak E.E. (1994). Normalization of CD4^+^ T-lymphocyte depletion in patients without HIV infection treated for tuberculosis. Chest.

[20] Kumarasamy C., Tiwary V., Sunil K., Suresh D., Shetty S., Muthukaliannan G.K., Baxi S., Jayaraj R. (2021). Prognostic Utility of Platelet-Lymphocyte Ratio, Neutrophil-Lymphocyte Ratio and Monocyte-Lymphocyte Ratio in Head and Neck Cancers: A Detailed PRISMA Compliant Systematic Review and Meta-Analysis. Cancers.

[21] Mazza M.G., Lucchi S., Rossetti A., Clerici M. (2020). Neutrophil-lymphocyte ratio, monocyte-lymphocyte ratio and platelet-lymphocyte ratio in non-affective psychosis: A meta-analysis and systematic review. World J. Biol. Psychiatry.

[22] Ji H., Li Y., Fan Z., Zuo B., Jian X., Li L., Liu T. (2017). Monocyte/lymphocyte ratio predicts the severity of coronary artery disease: A syntax score assessment. BMC Cardiovasc. Disord..

[23] Wang J., Zhu Q.W., Cheng X.Y., Liu J.Y., Zhang L.L., Tao Y.M., Cui Y.B., Wei Y. (2019). Assessment efficacy of neutrophil-lymphocyte ratio and monocyte-lymphocyte ratio in preeclampsia. J. Reprod. Immunol..

[24] Cheng H.R., Song J.Y., Zhang Y.N., Chen Y.B., Lin G.Q., Huang G.Q., He J.C., Wang Z. (2020). High Monocyte-To-Lymphocyte Ratio Is Associated With Stroke-Associated Pneumonia. Front. Neurol..

[25] Wang W., Wang L.F., Liu Y.Y., Yang F., Zhu L., Zhang X.H. (2019). Value of the Ratio of Monocytes to Lymphocytes for Monitoring Tuberculosis Therapy. Can. J. Infect. Dis. Med. Microbiol..

[26] Nishijima T.F., Muss H.B., Shachar S.S., Tamura K., Takamatsu Y. (2015). Prognostic value of lymphocyte-to-monocyte ratio in patients with solid tumors: A systematic review and meta-analysis. Cancer Treat. Rev..

[27] Alfhili M.A., Alsughayyir J., Basudan A., Ghneim H.K., Aboul-Soud M.A., Marie M., Dera A., Alfaifi M., Alkhathami A.G., Awan Z.A. (2022). Isolated and Combined Effect of Age and Gender on Neutrophil-Lymphocyte Ratio in the Hyperglycemic Saudi Population. Medicina.

[28] American Diabetes Association (2014). Standards of medical care in diabetes—2014. Diabetes Care.

[29] Alfhili M.A., Alsughayyir J., Basudan A.M., Ghneim H.K., Alfaifi M., Alamri H.S., Awan Z.A., Algethami M.R. (2022). Patterns of Dyslipidemia in the Anemic and Nonanemic Hypertensive Saudi Population: A Cross-Sectional Study. Int. J. Gen. Med..

[30] Allan C.A. (2014). Sex steroids and glucose metabolism. Asian J. Androl..

[31] Reinehr T., Roth C.L. (2018). Inflammation Markers in Type 2 Diabetes and the Metabolic Syndrome in the Pediatric Population. Curr. Diabetes Rep..

[32] Weyand C.M., Goronzy J.J. (2016). Aging of the Immune System. Mechanisms and Therapeutic Targets. Ann. Am. Thorac. Soc..

[33] Morbach H., Eichhorn E.M., Liese J.G., Girschick H.J. (2010). Reference values for B cell subpopulations from infancy to adulthood. Clin. Exp. Immunol..

[34] Liu S., Hempe J.M., McCarter R.J., Li S., Fonseca V.A. (2015). Association between Inflammation and Biological Variation in Hemoglobin A1c in U.S. Nondiabetic Adults. J. Clin. Endocrinol. Metab..

[35] Jaiswal A., Tabassum R., Podder A., Ghosh S., Tandon N., Bharadwaj D. (2012). Elevated level of C-reactive protein is associated with risk of prediabetes in Indians. Atherosclerosis.

[36] Simental-Mendía L.E., Lazalde B., Zambrano-Galván G., Simental-Saucedo L., Rábago-Sánchez E., Rodríguez-Morán M., Guerrero-Romero F. (2012). Relation between C-reactive protein and impaired fasting glucose in obese subjects. Inflammation.

[37] Al-Rubeaan K., Al-Manaa H., Khoja T., Ahmad N., Al-Sharqawi A., Siddiqui K., AlNaqeb D., Aburisheh K., Youssef A., Al-Batil A. (2014). The Saudi Abnormal Glucose Metabolism and Diabetes Impact Study (SAUDI-DM). Ann. Saudi Med..

[38] Mendes B.B., Oliveira A.C.R., Alcantara K.C. (2019). Comparison of the neutrophil-to-lymphocyte and platelet-to-lymphocyte ratios in normoglycemic and hyperglycemic subjects. Einstein (Sao Paulo).

[39] Demirtas L., Degirmenci H., Akbas E.M., Ozcicek A., Timuroglu A., Gurel A., Ozcicek F. (2015). Association of hematological indicies with diabetes, impaired glucose regulation and microvascular complications of diabetes. Int. J. Clin. Exp. Med..

[40] Kocak M.Z., Aktas G., Duman T.T., Atak B.M., Kurtkulagi O., Tekce H., Bilgin S., Alaca B. (2020). Monocyte lymphocyte ratio As a predictor of Diabetic Kidney Injury in type 2 Diabetes mellitus; The MADKID Study. J. Diabetes Metab. Disord..

[41] Huang Q., Wu H., Wo M., Ma J., Fei X., Song Y. (2020). Monocyte-lymphocyte ratio is a valuable predictor for diabetic nephropathy in patients with type 2 diabetes. Medicine (Baltimore).

[42] Wang H., Guo Z., Xu Y. (2022). Association of monocyte-lymphocyte ratio and proliferative diabetic retinopathy in the U.S. population with type 2 diabetes. J. Transl. Med..

[43] Huang Q., Wu H., Wo M., Ma J., Song Y., Fei X. (2021). Clinical and predictive significance of Plasma Fibrinogen Concentrations combined Monocyte-lymphocyte ratio in patients with Diabetic Retinopathy. Int. J. Med. Sci..

[44] Ilhan C., Citirik M., Uzel M.M., Kiziltoprak H., Tekin K. (2020). The usefulness of systemic inflammatory markers as diagnostic indicators of the pathogenesis of diabetic macular edema. Arq. Bras. De Oftalmol..

[45] Cho N.H., Shaw J.E., Karuranga S., Huang Y., da Rocha Fernandes J.D., Ohlrogge A.W., Malanda B. (2018). IDF Diabetes Atlas: Global estimates of diabetes prevalence for 2017 and projections for 2045. Diabetes Res. Clin. Pract..

[46] Tramunt B., Smati S., Grandgeorge N., Lenfant F., Arnal J.F., Montagner A., Gourdy P. (2020). Sex differences in metabolic regulation and diabetes susceptibility. Diabetologia.

[47] Anagnostis P., Christou K., Artzouchaltzi A.M., Gkekas N.K., Kosmidou N., Siolos P., Paschou S.A., Potoupnis M., Kenanidis E., Tsiridis E. (2019). Early menopause and premature ovarian insufficiency are associated with increased risk of type 2 diabetes: A systematic review and meta-analysis. Eur. J. Endocrinol..

[48] Beumer W., Gibney S.M., Drexhage R.C., Pont-Lezica L., Doorduin J., Klein H.C., Steiner J., Connor T.J., Harkin A., Versnel M.A. (2012). The immune theory of psychiatric diseases: A key role for activated microglia and circulating monocytes. J. Leukoc. Biol..

[49] Cai H.Q., Catts V.S., Webster M.J., Galletly C., Liu D., O’Donnell M., Weickert T.W., Weickert C.S. (2020). Increased macrophages and changed brain endothelial cell gene expression in the frontal cortex of people with schizophrenia displaying inflammation. Mol. Psychiatry.

[50] Palm M., Axelsson O., Wernroth L., Larsson A., Basu S. (2013). Involvement of inflammation in normal pregnancy. Acta Obstet. Gynecol. Scand..

[51] Liu L., Zhao G., Fan H., Zhao X., Li P., Wang Z., Hu Y., Hou Y. (2014). Mesenchymal stem cells ameliorate Th1-induced pre-eclampsia-like symptoms in mice via the suppression of TNF-alpha expression. PLoS ONE.

[52] Wang J., Zhu Q.W., Cheng X.Y., Sha C.X., Cui Y.B. (2020). Clinical significance of neutrophil-lymphocyte ratio and monocyte-lymphocyte ratio in women with hyperglycemia. Postgrad. Med..

[53] Chen H., Li M., Liu L., Dang X., Zhu D., Tian G. (2019). Monocyte/lymphocyte ratio is related to the severity of coronary artery disease and clinical outcome in patients with non-ST-elevation myocardial infarction. Medicine (Baltimore).

[54] Mirna M., Schmutzler L., Topf A., Hoppe U.C., Lichtenauer M. (2021). Neutrophil-to-lymphocyte ratio and monocyte-to-lymphocyte ratio predict length of hospital stay in myocarditis. Sci. Rep..

[55] Aydin C., Engin M. (2021). The Value of Inflammation Indexes in Predicting Patency of Saphenous Vein Grafts in Patients With Coronary Artery Bypass Graft Surgery. Cureus.

[56] Kadiyoran C., Zengin O., Cizmecioglu H.A., Tufan A., Kucuksahin O., Cure M.C., Cure E., Kucuk A., Ozturk M.A. (2019). Monocyte to Lymphocyte Ratio, Neutrophil to Lymphocyte Ratio, and Red Cell Distribution Width are the Associates with Gouty Arthritis. Acta Med..

[57] Buttle T.S., Hummerstone C.Y., Billahalli T., Ward R.J., Barnes K.E., Marshall N.J., Spong V.C., Bothamley G.H. (2021). The monocyte-to-lymphocyte ratio: Sex-specific differences in the tuberculosis disease spectrum, diagnostic indices and defining normal ranges. PLoS ONE.

[58] Long V.S., Ngiam J.N., Chew N., Tham S.M., Lim Z.Y., Li T., Cen S., Annadurai J.K., Thant S.M., Tambyah P.A. (2021). Haematological profile of COVID-19 patients from a centre in Singapore. Hematology.

[59] Muto R., Kato S., Lindholm B., Qureshi A.R., Ishimoto T., Kosugi T., Maruyama S. (2022). Increased Monocyte/Lymphocyte Ratio as Risk Marker for Cardiovascular Events and Infectious Disease Hospitalization in Dialysis Patients. Blood Purif..

[60] Li Y., Liu Y., Zhou C., Zhang Z., Zuo X., Li J., Cao Q. (2021). Monocyte/lymphocyte ratio as a predictor of 30-day mortality and adverse events in critically ill patients: Analysis of the MIMIC-III database. Zhonghua Wei Zhong Bing Ji Jiu Yi Xue.

[61] Kamiya N., Ishikawa Y., Kotani K., Hatakeyama S., Matsumura M. (2022). Monocyte-to-Lymphocyte Ratio in the Diagnosis of Lymphoma in Adult Patients. Int. J. Gen. Med..

[62] Xiang J., Zhou L., Li X., Bao W., Chen T., Xi X., He Y., Wan X. (2017). Preoperative Monocyte-to-Lymphocyte Ratio in Peripheral Blood Predicts Stages, Metastasis, and Histological Grades in Patients with Ovarian Cancer. Transl. Oncol..

[63] Soibi-Harry A.P., Amaeshi L.C., Garba S.R., Anorlu R.I. (2021). The relationship between pre-operative lymphocyte to monocyte ratio and serum cancer antigen-125 among women with epithelial ovarian cancer in Lagos, Nigeria. Ecancermedicalscience.

[64] Adamkiewicz M., Bryniarski P., Kowalik M., Burzynski B., Rajwa P., Paradysz A. (2021). Lymphocyte-to-Monocyte Ratio Is the Independent Prognostic Marker of Progression in Patients Undergoing BCG-Immunotherapy for Bladder Cancer. Front. Oncol..

[65] D’Andrea D., Moschini M., Gust K.M., Abufaraj M., Özsoy M., Mathieu R., Soria F., Briganti A., Rouprêt M., Karakiewicz P.I. (2017). Lymphocyte-to-monocyte ratio and neutrophil-to-lymphocyte ratio as biomarkers for predicting lymph node metastasis and survival in patients treated with radical cystectomy. J. Surg. Oncol..

[66] Zhang G.M., Zhu Y., Luo L., Wan F.N., Zhu Y.P., Sun L.J., Ye D.W. (2015). Preoperative lymphocyte-monocyte and platelet-lymphocyte ratios as predictors of overall survival in patients with bladder cancer undergoing radical cystectomy. Tumour Biol..

[67] Yoshida T., Kinoshita H., Yoshida K., Mishima T., Yanishi M., Inui H., Komai Y., Sugi M., Inoue T., Murota T. (2016). Prognostic impact of perioperative lymphocyte-monocyte ratio in patients with bladder cancer undergoing radical cystectomy. Tumour Biol..

[68] Chen L., Zhang F., Sheng X.G., Zhang S.Q. (2015). Decreased pretreatment lymphocyte/monocyte ratio is associated with poor prognosis in stage Ib1-IIa cervical cancer patients who undergo radical surgery. OncoTargets Ther..

[69] Hu P., Shen H., Wang G., Zhang P., Liu Q., Du J. (2014). Prognostic significance of systemic inflammation-based lymphocyte- monocyte ratio in patients with lung cancer: Based on a large cohort study. PLoS ONE.

[70] Kang Y., Zhu X., Lin Z., Zeng M., Shi P., Cao Y., Chen F. (2021). Compare the Diagnostic and Prognostic Value of MLR, NLR and PLR in CRC Patients. Clin. Lab..

[71] Xu Z., Zhang J., Zhong Y., Mai Y., Huang D., Wei W., Huang J., Zhao P., Lin F., Jin J. (2021). Predictive value of the monocyte-to-lymphocyte ratio in the diagnosis of prostate cancer. Medicine (Baltimore).

[72] Wan H., Cai Y., Wang Y., Fang S., Chen C., Chen Y., Xia F., Wang N., Guo M., Lu Y. (2020). The unique association between the level of peripheral blood monocytes and the prevalence of diabetic retinopathy: A cross-sectional study. J. Transl. Med..

